# Concurrent chemoradiotherapy was associated with a higher severe late toxicity rate in nasopharyngeal carcinoma patients compared with radiotherapy alone: a meta-analysis based on randomized controlled trials

**DOI:** 10.1186/s13014-015-0377-9

**Published:** 2015-03-26

**Authors:** Cheng-run Du, Hong-mei Ying, Fang-fang Kong, Rui-ping Zhai, Chao-su Hu

**Affiliations:** Department of Radiation Oncology, Fudan University Shanghai Cancer Center, 270 Dongan Road, 200032 Shanghai, People’s Republic of China

**Keywords:** Nasopharyngeal carcinoma, Concurrent chemoradiotherapy, Severe late toxicities, Meta-analysis

## Abstract

**Background:**

To investigate the incidence and risk of severe late toxicity with concurrent chemoradiotherapy (CCRT) in nasopharyngeal carcinoma patients.

**Methods:**

Eligible studies included prospective randomized controlled trials (RCTs) evaluating CCRT versus radiotherapy alone in patients with nasopharyngeal carcinoma and in which data on severe late toxicities were available. Random effects or fixed effect models were applied to obtain the summary incidence, relative risks (RRs) and 95% confidence intervals (CIs).

**Results:**

Five RCTs with 1102 patients with NPC were included in this analysis. The summary incidence of overall severe late toxicities in patients receiving CCRT was 30.7% (95% CI, 18–47.2%) and the incidence of radiotherapy alone group was 21.7% (95% CI, 13.3–33.4%). The use of concurrent chemotherapy was associated with an increased risk of severe late toxicities, with a RR of 1.349 (95% CI, 1.108–1.643; P = 0.005). As for specific late toxicity, CCRT significantly increased the risk of ear deafness/otitis (RR = 1.567; 95% CI, 1.192–2.052), but other late toxicities were not significantly different. Patients receiving concurrent chemotherapy regimens with 3-week high-dose cisplatin (HC) have a higher risk of ear deafness/otitis (RR = 1.672; 95% CI, 1.174–2.382; P = 0.026). However, there was no significant increase in the RR of severe ear complication with the addition of non-3-week high-dose cisplatin (nonHC) regimens (RR = 1.433; 95% CI, 0.946–2.171; P = 0.095).

**Conclusion:**

With the present evidence, the addition of concurrent chemotherapy seems to increase the risk of severe late toxicities in patients with NPC, especially when using HC regimen for the occurrence of severe ototoxicity.

## Background

Nasopharyngeal carcinoma (NPC) is sensitive to radiation, but the outcomes of patients with advanced-stage NPC were unsatisfactory [1,2]. Given the chemosensitive nature of NPC, the combination of chemotherapy and radiation has been investigated for advanced NPC. No consensus on the optimal protocol had been reached until the Intergroup 0099 study [3], which demonstrated that concurrent chemoradiotherapy (CCRT) plus adjuvant chemotherapy using cisplatin-based chemotherapy was superior to radiation therapy alone. Subsequently, evidence increasingly mounted from randomized trials [4-8] and meta-analyses [9] confirming the advantage of CCRT.

Although the superiority of CCRT for NPC has been indisputable, there are concerns about the side effects caused by the combination of concurrent chemotherapy and radiotherapy. Severe acute toxicities could impair the compliance of treatment. Severe late toxicities (grade 3 or 4) can be life-threatening or significantly erode the patient’s quality of life (QoL) and functional status [10]. As the overall survival for NPC patients improves, late toxicities may become more frequent. Concurrent chemotherapy was found to increase the acute toxicities during radiotherapy [6-8]. However, the overall incidence and risk of severe late toxicities with additional concurrent chemotherapy in NPC remain unknown because many of the published data were based on studies that were either retrospective, or with inadequate duration of observation for full assessment of long-term toxicity, or were obscured by treatment heterogeneity [11-13].

We conducted this meta-analysis of prospective randomized controlled trials (RCTs) reporting severe late toxicities to investigate the overall incidence and risk of severe late toxicities with CCRT in NPC to help clinicians target possible severe late effects that need special attention during patient follow-up.

## Methods

We identified and selected relevant studies by searching the databases: Web of knowledge, Embase, Cochrane library databases and Pubmed updated to August 2014. The search was conducted by using the keywords “nasopharyngeal carcinoma”, “radiotherapy”, “chemotherapy”, and “chemoradiotherapy” and was limited to human studies and clinical trials published in English. The reference list of each article obtained was checked for further potential studies. Only RCTs in which CCRT was compared with radiotherapy alone in NPC patients and the detailed data on severe late toxicities were reported, were eligible for inclusion in the meta-analysis. The report quality of the clinical trials was assessed and calculated using Jadad scale including randomization, double-blinding, and withdrawals as previously described [14].

Data from the studies were extracted independently by two investigators (CD and FK) using standardized data forms and disagreement was resolved by consensus. The following information was extracted: author’s name, publication year, number of enrolled subjects, treatment arms, number of patients in each group, the duration of follow-up and events of severe late toxicities. This study was performed with the approval of Institutional Review Boards of Fudan University Shanghai Cancer Center.

### Statistical analysis

The pooled analysis of incidence and RR were performed with Open Meta-Analyst software version 3.13. The publication bias was tested with Stata/SE 12.0. A classic half-integer continuity correction was applied to obtain the relative risk (RR) and variance for studies reporting zero events in a treatment or control arm. Between-study heterogeneity was estimated using chi-squared and I^2^ tests. Heterogeneity was considered statistically significant when P < 0.05 or I^2^ > 50%. If heterogeneity existed, data was analyzed using a random effects model. In the absence of heterogeneity, a fixed effects model was used. The RR was considered significant when P value was less than 0.05 (2-tailed), with values of <1 favoring concurrent chemoradiotherapy. The presence of publication bias was assessed with the Begg’s [15] and Egger’s tests [16].

## Results

### Patients characteristics and quality assessment

**Figure 1:**
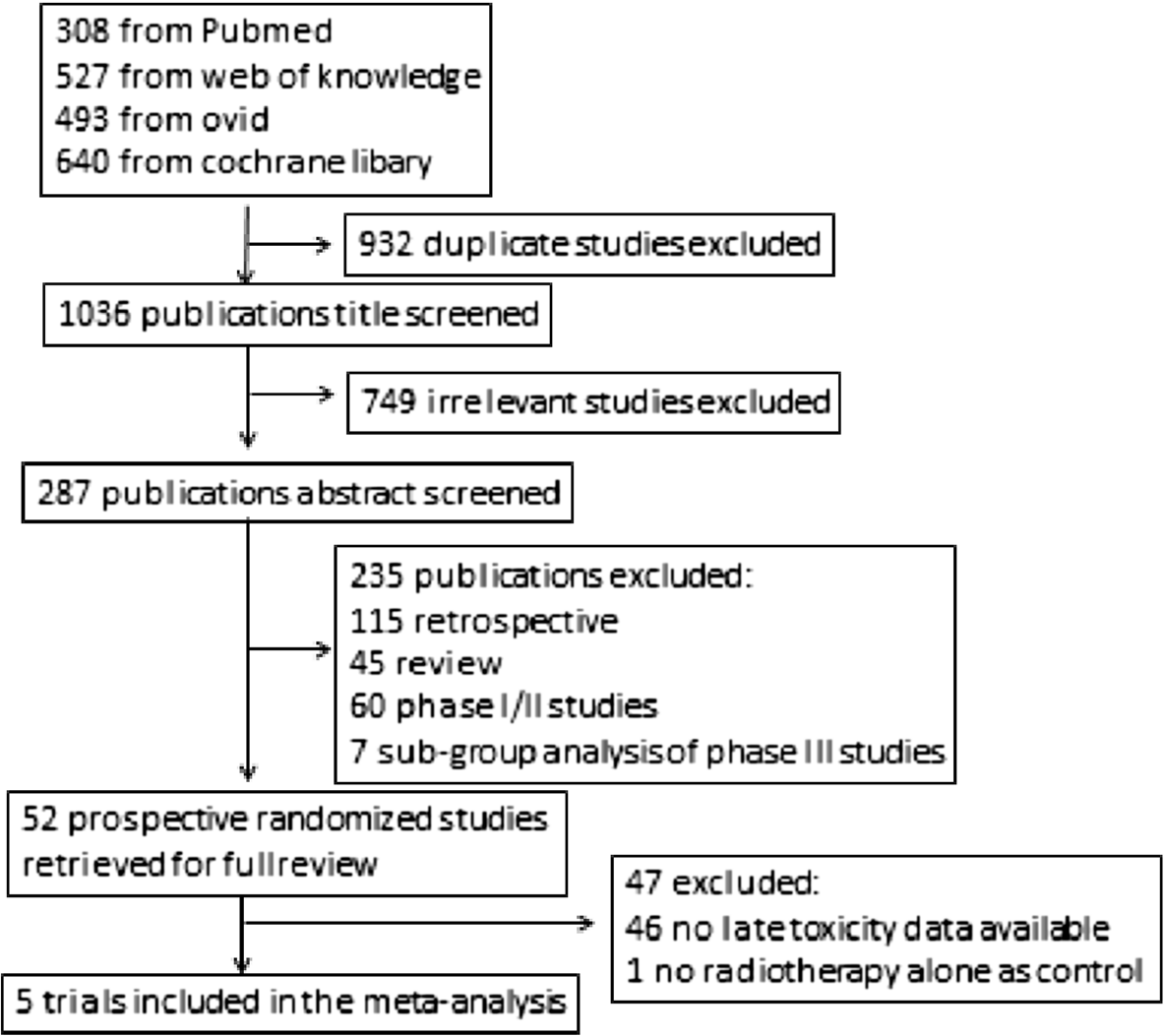
**CONSORT diagram: flow chart of trail selection process for the meta-analysis.**

Through the electronic databases, a total of 1968 citations were searched. Initially, 932 studies were excluded for duplicate. After screening through titles and abstracts, a total of fifty-two randomized controlled trials (RCTs) investigating CCRT versus radiotherapy in patients with NPC were identified. The data on the severe late toxicity were reported in 5 studies [17-21], which were further included in the meta-analysis. Figure 1 shows the selection process in detail.

The main characteristics of the included studies are presented in Table 1. Patients in Lee’s trial [18] were randomized to receive conventional-fractionation RT alone (CF group), accelerated-fractionation RT alone (AF group), conventional-fractionation RT plus concurrent-adjuvant chemotherapy (CF + C group), or accelerated-fractionation RT plus concurrent-adjuvant chemotherapy (AF + C group). Only the patients in CF group and CF + C group were included in our analysis. The included RCTs had a median follow-up of at least 5 years. A total of 1102 patients with histologically proven NPC were available for the meta-analysis. There were 547 patients in CCRT group and 555 in RT group. Late toxicity in all the included trials was graded according to Late Radiation Morbidity Scoring Criteria of the Radiation Therapy Oncology Group [22]. Severe late toxicity was defined as grade 3, 4 and 5 taken together.

**Table 1:** **Characteristics of RCTs Included in the meta-analysis**

**Study**	**No. of patients**	**Inclusion period**	**Stage**	**Median follow-up (Ms)**	**Group**	**Radiotherapy**	**CCRT**	**AC**
Lee et al. (2011) [19]	93	1999-2004	AJCC stage T3-4 N0-1 M0	75	CCRT + AC	≥66 Gy (2 Gy/Fx/d, 5Fx/wk)	Cisplatin 100 mg/m2 intravenously every 3 weeks	Cisplatin 80/m2 + fluorouracil 1000 mg/m2/day by 96-h infusion every 4 weeks for 3 cycles.
					RT			
Lee et al. (2010) [21]	348	1999–2004	AJCC stage III and IV, any T, N2 or N3, M0	71	CCRT + AC	≥66 Gy (2 Gy/Fx/d, 5Fx/wk)	Cisplatin 100 mg/m2 every 3wks on days 1,22,43	Cisplatin 80 mg/m2 + fluorouracil 1000 mg/m2/d every 4 wks on days 71,99 and 127
					RT			
Chen et al. (2013) [18]	316	2002-2005	AJCC stage III and IVb	70	CCRT + AC	≥66 Gy (2 Gy/Fx/d, 5Fx/wk)	Cisplatin 40 mg/m2 on day 1 weekly	Cisplatin 80 mg/m2 on day 1 and fluorouracil 800 mg/m2 daily as an intravenous, 120-hour infusion) on days 1 through 5
					RT			
Chen et al. (2011) [20]	230	2003-2007	Chinese 1992 staging system stage II	60	CCRT	68–70 Gy (2Gy/Fx/d, 5Fx/wk)	Cisplatin 30 mg/m2 weekly	none
					RT			
Wu et al. (2013) [17]	115	2001-2003	AJCC stage III and IVb	114	CCRT	70-74Gy (2Gy/Fx/d, 5Fx/wk)	Oxaliplatin70 mg/m2 on days 1 every week	none
					RT			

All the included trials reported withdrawals and drop-outs. Four trials had mentioned the concealment of allocation clearly in the randomization process and had Jadad scores of 3. One trial did not mention the method of generating sequence of randomization and had Jadad scores of 2.

### Incidence and RR of overall severe late toxicities

**Figure 2:**
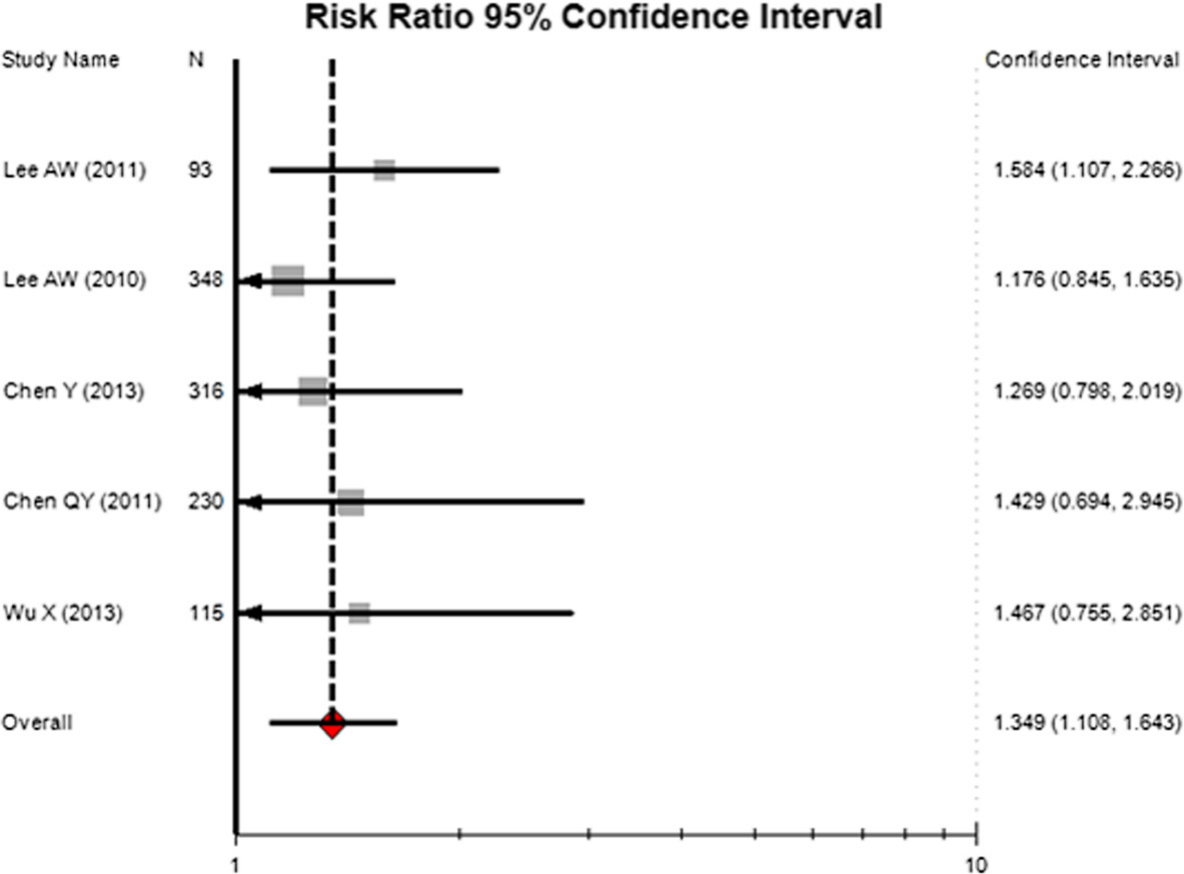
**Relative risk of severe late toxicities with the addition of concurrent chemotherapy.**

For total types of severe late toxicities, there were 150 events among 547 NPC patients that received CCRT, conferring a summary incidence of 30.7% (95% CI, 18–47.2%; heterogeneity test: P < 0.001; I^2^ = 47.7%). There were 118 events among 555 patients receiving RT alone, with an incidence of 21.7% (95%CI, 13.3–33.4%; heterogeneity test: P < 0.001; I^2^ = 46.3%). The summary RR of overall severe late toxicities for CCRT versus RT was 1.349 (95% CI, 1.108–1.643; P = 0.005) (Figure 2), suggesting a significant increase of severe late toxicities in NPC patients receiving CCRT. No significant heterogeneity was observed (Q = 1.592; P = 0.81; I^2^ = 0.0%).

### Incidence and RR of specific late toxicity

The pooled analysis of the incidence and RR of specific late toxicities recorded by at least 2 trials is shown in Table 2. The main severe late toxicities included: ear deafness/otitis, cranial neuropathy, neck soft tissue damage, endocrine dysfunction. CCRT significantly increased the risk of ear deafness/otitis compared with RT (RR = 1.567; 95% CI, 1.192–2.052), but no significant difference was observed in the risk of other kinds of severe late toxicities. The included trials were stratified for concurrent chemotherapy regimen. Patients receiving concurrent chemotherapy regimens with 3-week high-dose cisplatin (HC) have a higher risk of ear deafness (RR = 1.672; 95% CI, 1.174–2.382; P = 0.026). However, there was no significant increase in the RR of severe ototoxicity with the addition of non-3-week high-dose cisplatin (nonHC) regimens (RR = 1.433; 95% CI, 0.946–2.171; P = 0.095) (Table 3).

**Table 2:** **Incidence and relative risk (RR) of specific late toxicity**

**Late toxicities**	**No. of studies**	**No. of severe late toxicity/total no. of patients**		**Incidence of severe late toxicity, % (95% CI)**		**RR**
		**Chemoradiotherapy**	**Radiotherapy**	**Chemoradiotherapy**	**Radiotherapy**	
Overall late toxicities	5	150/547	118/555	30.7(18-47.2)	21.7(13.3-33.4)	1.349(1.108-1.619)
Ear (deafness/otitis)	5	107/547	72/555	20.7(12-33.2)	13.3(9.3-18.7)	1.567(1.197-2.052)
Cranial neuropathy	4	19/431	21/441	5.5(3.6-8.5)	5.2(3.4-7.9)	0.923(0.351-2.427)
Soft tissue damage at neck	4	17/431	17/441	4.6(2.9-7.2)	4.2(2.6-6.6)	1.007(0.522-1.94)
Peripheral neuropathy	3	6/372	2/385	1.8(0.9-3.9)	1.6(0.5-5)	2.295(0.41-12.845)
Temporal lobe necrosis	2	5/200	11/209	2.9(1.3-6.5)	5.8(3.2-10.2)	0.47(0.079-2.809)
Brachial plexopathy	2	0/214	1/227	0.6(0.1-4.1)	0.7(0.1-3.3)	0.567(0.048-6.707)
Endocrine dysfunction	2	15/214	12/227	7.1(4.3-11.4)	5.3(3.1-9.2)	1.312(0.629-2.74)
Visual toxicity	2	3/200	3/209	2.4(0.8-7.2)	2.1(0.7-6.4)	1.14(0.236-5.507)
Dysphagia	2	2/214	0/227	1.2(0.3-3.9)	0.5(0.1-3.6)	2.973(0.272-32.51)
Bone necrosis	2	2/330	1/334	0.6(0.2-2.4)	0.5(0.1-2.3)	1.622(0.201-13.111)
Mucosal damage	2	4/231	1/232	2.1(0.8-5.1)	0.7(0.1-3.2)	2.891(0.431-19.409)
Radiation-induced malignancy	2	0/330	1/333	0.3(0-2.1)	0.5(0.1-2.3)	0.524(0.044-6.226)

**Table 3:** **Relative risk of severe ototoxicity by concurrent chemotherapy regimen**

**Study**	**No. of events/patient size**		**Relative risk**	**95% CI**	**P value**
	**CCRT**	**RT**			
HC					
Lee et al. (2011) [19]	22/42	12/51	2.226	1.256 to 3.947	
Lee et al. (2010) [21]	37/172	27/176	1.402	0.895 to 2.198	
Overall			***1.672***	***1.174 to 2.382***	***0.026***
Non-HC					
Chen et al. (2013) [18]	28/158	18/158	1.556	0.898 to 2.695	
Chen et al. (2011) [20]	12/116	8/114	1.474	0.626 to 3.471	
Wu et al. (2013) [17]	8/59	11/56	1.085	0.421 to 2.794	
Ovarall			***1.433***	***0.946 to 2.171***	***0.095***

CCRT: concurrent chemoradiotherapy; RT: radiotherapy; HC: 3-week high-dose cisplatin; non-HC: non-3-week high-dose cisplatin.

### Publication bias

There was no evidence of publication bias for RR of overall severe late toxicities by either the Begg’s or the Egger’s test (Begg’s P = 0.806 and Egger’s P = 0.687). No publication bias for RR of severe deafness/otitis was observed (Begg’s P = 0.806 and Egger’s P = 0.696).

## Discussion

As far as we know, this is the first meta-analysis evaluating the incidence and risk of severe late toxicities associated with CCRT. In current study, we reviewed the existing data on the severe late toxicities from RCTs in NPC patients by a meta-analytical method. In this analysis, all the included RCTs have a median follow-up of at least 5 years, which was considered to be a useful starting point for reporting late toxicity. It’s revealed in our analysis that NPC patients treated with CCRT had a significantly increased risk of severe late toxicities compared with those given with RT alone. The incidence of severe late toxicities for patients receiving CCRT was 30.7% compared with 21.7% for those treated with RT. Whether the addition of concurrent chemotherapy increases the incidence and severity of late toxicities in NPC patients was controversial in the literature. These findings could be explained by that the published data on late toxicities induced by CCRT were usually from studies that are small sample sizes, retrospective, involved treatment heterogeneity, or with inadequate follow-up [10-12].

Ototoxicity is the most common severe late toxicity, accounting for almost two thirds of total severe late toxicities in this analysis. CCRT incurred a significant 1.567-fold increase in the risk of severe ototoxicity, which is not surprising because both cisplatin and RT are known to cause hearing loss. However, with weekly intermediate dose of cisplatin or oxaliplatin (nonHC), the patients didn’t experience a statistically significant increased risk. No additive effect for weekly intermediate dose of cisplatin on ototoxicity may be explained by that the dose level of cisplatin given per infusion was too low to induce ototoxicity by itself [23]. Although HC regimens had resulted in favorable outcome for locally advanced NPC, it was associated with increased acute and poor compliance. In the Intergroup 0099 study, only 63% of patients who were scheduled to receive three courses of concurrent 100 mg/m2 cisplatin actually did so. This issue may be overcome by the change of combination schedule of cisplatin or the use of other anticancer agents. It’s reported [24] that a weekly intermediate dose of cisplatin could decrease interruptions in radiation treatment and reduce acute toxic effects without compromising local control. Weekly oxaliplatin combined concomitantly with RT was also found to achieve a significant improvement in therapeutic outcome with minimal and acceptable additional toxic effects [14]. Thus, nonHC regimens may be an alternative of treatment for NPC in order to reduce the rate of ototoxicity.

Except for ototoxicity, the risk of other severe late toxicities didn’t increase with the addition of concurrent chemotherapy in this analysis, for which we suggest several possible explanations: the small number of events recorded; underreporting of rare adverse events; the fact that clinical trials are usually not designed specifically to address toxic events; and the small number of randomized controlled trials included.

There are some limitations in this meta-analysis. First, the follow-up duration of the included trials was inadequate for assessing all types of late toxicities. Five year follow-up is long enough to assess the development of ototoxicity but may be inadequate for neurological complications, the median latency of which exceeds 5 years. Kong et al. [25] reported that the occurrence of cranial neurophathy increased with prolonged follow-up, reaching 44.5% at 20 years. But the included data in this analysis is the most updated data we can get from RCTs. This limitation can only be overcome by the further update of the RCTs. Second, the number RTCs included in this analysis is small. We have searched the Web of knowledge, Embase, Cochrane controlled trials register and Pubmed updated to August 2014, but only five RCTs fulfilled the inclusion criteria. The data on severe late toxicities are unavailable in most of RCTs. Thus the updates of long term results from RCTs are encouraged to provide a better understanding of the potential adverse impacts of CCRT on NPC patients. Third, this meta-analysis is based on published data and tends to overestimate treatment effects compared with individual patient data analyses.

## Conclusion

This study demonstrates that the use of CCRT seems to increase the risk of severe late toxicities in patients with NPC, especially when using HC regimen for the occurrence of severe ototoxicity. But one should be cautious when interpreting these results due to the limitations of our study. Additionally, as concurrent chemotherapy with cisplatin gains greater clinical use, clinicians should be aware of the risks of severe late toxicities with the addition of high dose cisplatin and adequately explain these risks to gain truly informed consent of our patients.

## Abbreviations

NPC: Nasopharyngeal carcinoma
CCRT: Concurrent chemoradiotherapy
QoL: Quality of life
RR: Relative risk
RCTs: Randomized controlled trials
HC: 3-week high-dose cisplatin
nonHC: Non-3-week high-dose cisplatin
